# Isolation of the Novel Phage PHB09 and Its Potential Use against the Plant Pathogen *Pseudomonas syringae* pv. *actinidiae*

**DOI:** 10.3390/v13112275

**Published:** 2021-11-14

**Authors:** Yanxi Liu, Mengjiao Liu, Ran Hu, Jun Bai, Xiaoqing He, Yi Jin

**Affiliations:** College of Biological Sciences and Biotechnology, Beijing Forestry University, Beijing 100083, China; liuyanxi1206@163.com (Y.L.); liumengjiao_97@163.com (M.L.); hurannn@163.com (R.H.); 15637165241@163.com (J.B.); lenahe@bjfu.edu.cn (X.H.)

**Keywords:** bacteriophage, phage therapy, *Pseudomonas syringae* pv. *actinidiae*, *Caudoviricetes*

## Abstract

Bacteriophages are viruses that specifically infect target bacteria. Recently, bacteriophages have been considered potential biological control agents for bacterial pathogens due to their host specificity. *Pseudomonas syringae* pv. *actinidiae* (Psa) is a reemerging pathogen that causes bacterial canker of kiwifruit (*Actinidia* sp.). The economic impact of this pest and the development of resistance to antibiotics and copper sprays in Psa and other pathovars have led to investigation of alternative management strategies. Phage therapy may be a useful alternative to conventional treatments for controlling Psa infections. Although the efficacy of bacteriophage φ6 was evaluated for the control of Psa, the characteristics of other DNA bacteriophages infecting Psa remain unclear. In this study, the PHB09 lytic bacteriophage specific to Psa was isolated from kiwifruit orchard soil. Extensive host range testing using Psa isolated from kiwifruit orchards and other *Pseudomonas* strains showed PHB09 has a narrow host range. It remained stable over a wide range of temperatures (4–50 °C) and pH values (pH 3–11) and maintained stability for 50 min under ultraviolet irradiation. Complete genome sequence analysis indicated PHB09 might belong to a new myovirus genus in *Caudoviricetes*. Its genome contains a total of 94,844 bp and 186 predicted genes associated with phage structure, packaging, host lysis, DNA manipulation, transcription, and additional functions. The isolation and identification of PHB09 enrich the research on *Pseudomonas* phages and provide a promising biocontrol agent against kiwifruit bacterial canker.

## 1. Introduction

*Pseudomonas syringae* is a species complex of bacterial plant pathogens infecting more than 180 plant species [1], including phytopathogens that have significant effects on the agriculture sector by causing severe economic losses worldwide. Many globally important crops fall within the host range of *P. syringae*, making this group one of the most economically destructive pathogens [2]. Some kiwifruit cultivars, especially red-fleshed kiwifruit “Hongyang” and “Enza-Red” (*Actinidia chinensis* var. *chinensis*), are highly susceptible to *P. syringae* pv. *actinidiae* (Psa) [3,4], with infection causing bacterial canker and severe economic losses in many countries [5,6]. In recent studies, Psa can be divided into at least six different biovars [7]. Biovar 1 is associated with outbreaks of the disease in Japan and Italy [8]. Biovar 2 has only been reported in South Korea [9]. Biovar 3 derived from a Psa population from China, is responsible for substantial economic losses worldwide [10]. Biovar 4 is an avirulent strain [11], and Biovars 5 and 6 are identified in Japan [12,13]. Copper pesticide combined with antibiotics and strict orchard management have been used to control the disease and spread of Psa [14]. However, these strategies are not completely effective, and their widespread use can induce resistance in the pathogen and further environmental damage [15]. Therefore, a phage-based alternative strategy is required.

Bacteriophages, viruses that specifically infect target bacteria, are one of the most abundant types of organisms in all environments. Approximately 10^30–31^ bacteriophages (phages) are present in the biosphere. Bacteriophages are relatively safe because they exert no activity against animal or plant cells and do not affect other beneficial microorganisms out of the host range [16]. Phages are promising tools to combat microbial resistance and can be used in medicine, agriculture, and related fields [17] to counter the development of multidrug-resistant bacteria, which pose a real threat to the control of infectious diseases globally. Currently, several bacteriophages have been characterized and investigated as agents for phage therapy in diseases caused by plant-pathogenic bacteria [18], including *Dickeya solani* [19], *Pectobacterium carotovorum* [20], and *Ralstonia solanacearum* [21]. Some phage-based products are already approved for agricultural use [e.g., EcoShield™ from Intralytix Inc. (Baltimore, MD, USA), Agriphage™ from Omnilytics (Salt Lake City, UT, USA), and Phagelux Inc. (Shanghai, China)] [22].

Recently, bacteriophages of Psa have been reported and characterized, but only a few of them showed control efficacy on bacterial canker [23]. The dsRNA phage φ6, a well-studied phage was reported to control infection of plant-pathogenic *P. syringae* pathovars ex vivo [1]. However, resistance to phage φ6 in plants is a problem. According to several reports, such resistance can be overcome using phage cocktails containing novel phages [24]. Meanwhile, some lytic DNA phages infect Psa also showed strong potential against kiwifruit canker. *Podoviridae* phages CHF1, CHF7, CHF19 and CHF21 showed the ability of reducing Psa load and leaf damages under greenhouse conditions [25]. *Myoviridae* phage PPPL-1 isolated from orchard soil in Korea could efficiently control bacterial canker in kiwifruit [11,26]. These findings suggest that bacteriophage is a promising alternative for the biocontrol of Psa. Most bacteriophages effective against plant-pathogenic bacteria belong to the order *Caudovirales*, which is composed of three families: *Myoviridae*, *Siphoviridae*, and *Podoviridae* [27]. Although some Psa phages have been isolated and characterized [11,28], none are yet approved for the biocontrol of Psa-induced kiwifruit canker. Use of phage therapy against Psa remains in the early stages [29], and novel phages are needed for this purpose. In this study, we isolated and characterized a novel DNA phage belonging to *Caudoviricetes* that efficiently infected Psa, with the long-term goal of testing and developing a phage-based biocontrol solution. 

## 2. Materials and Methods

### 2.1. Bacterial Strains and Culture Conditions

The bacterial strain PsaBJ530 (CGMCC1.19157) used in this study was isolated previously from the branches of canker-infected, red-fleshed kiwifruit “Hongyang” cultivar in Cangxi County, Sichuan Province, and its pathogenicity has been confirmed in vivo (data not shown). The biovar of PsaBJ530 was determined by PCR using primer CAGGAATTCATGACTTCTCA and TAGTCTCGAAGATTCAATGG. Preservation of the strain was achieved using 15% glycerol stocks stored at − 80 °C. PsaBJ530 was subjected to culturing at 25 °C with shaking in tryptic soy broth (TSB) prior to phage infection. 

### 2.2. Phage Isolation and Purification

Soil samples were collected from “Hongyang” cultivar kiwifruit orchards in Sichuan Province and used to screen phages specific to the isolated bacterial strain PsaBJ530. For phage isolation, 2 g soil was placed in a 50-mL Falcon tube containing 20 mL TSB and 4 sterile tungsten beads (Qiagen, Manchester, UK). The tube was vortexed for 5 min and centrifuged at 4500 rpm for 5 min. A 5-mL aliquot of the resulting supernatant was filtered through a 0.22-µm filter (Millipore, Sigma-Aldrich, Gillingham, UK) to remove any bacteria. The optical density (OD) of overnight bacterial cultures were measured at 600 nm and adjusted to an optical density of 0.8. Then, 100 µL of the bacterial host and 2 mL of a serially diluted environmental sample were added to a soft top agar overlay and incubated overnight at 25 °C. Three successive single-plaque isolations were performed to achieve pure phage isolates. The phage stocks were stored at 4 °C until use. DNA integrity and size distribution were assessed on a 1% (*w/v*) agarose gel and visualized with ethidium bromide [30].

### 2.3. Transmission Electron Microscopy

Transmission electron microscopy was used for determination of phage morphology. A filtered high-titer (~10^11^ PFU/mL) phage stock solution was placed on the surface of carbon-coated copper grids (Agar Scientific, Essex, UK). The phage was negatively stained using 2% (*w/v*) uranyl acetate, which was removed after 2 min. The grids with adsorbed phages were air dried, and the morphology of the phage was observed by transmission electron microscopy (Hitachi H-7650, Tokyo, Japan) at an acceleration voltage of 80 kV using a charge-coupled device camera (AMT400, Woburn, MA, USA).

### 2.4. Determination of the Optimal Multiplicity of Infection and One-Step Growth Curve

The double agar overlay plaque assay was used with some modifications to determine phage titer. Briefly, 100 μL serially diluted phage culture was mixed with 100 μL PsaBJ530 cells (early log phase, OD_600_ 0.8). This mixture was added to 10 mL top agar (TSB with 0.7% agar), poured onto a plate (TSB with 1.5% agar), and incubated at 25 °C for 12–16 h to form phage plaques. Three parallel assessments were performed.

To determine the optimal multiplicity of infection (MOI), exponential-phase Psa cells were diluted to 10^9^ CFU/mL. Phage solution (1 mL) was added to 1 mL of diluted bacterial cells at ratios of 1000, 100, 10, 1, 0.1, 0.01, 0.001, and 0.0001, respectively, and the mixture was shaken at 25 °C for 4 h at 200 rpm. Subsequently, the culture was centrifuged at 12,000× *g* for 10 min to remove bacterial cells, and the supernatant was filtered through a 0.22-μm pore-size membrane filter. The titer of the phage filtrate was determined using the double agar overlay plaque assay, as described previously. The dilution that generated the highest phage titer was considered the optimal MOI. Three parallel assessments were performed. 

In the one-step growth curve assay, 15 mL bacterial cells (10^9^ CFU/ mL) were mixed with 15 mL phage solution at an MOI of 0.001. The mixture was incubated at 25 °C for 5 min and centrifuged at 12,000× *g* for 30 s to remove unabsorbed free phages. The precipitate was suspended in TSB after two washes and then mixed with 30 mL TSB, followed by shaking at 25 °C and 180 rpm. This time was defined as t = 0 and subsequent time points as t = 15, 30, 45, 60, 75, 90, 105, 120, 135, and 150 min; at each time point, we collected 500 μL samples. The phage titer was determined using the double-layer agar method. The experiment was repeated three times, and three parallel tests were performed to measure phage titers at each time point.

### 2.5. Phage Host Range

The host range of phage PHB09 was investigated via infection of 22 bacterial strains using the spot assay method. Six biovar 2/3 Psa strains, including BJ530, BJ9, and BST isolated from kiwifruit orchards in Sichuan Province and three Psa strains from the Korean Agriculture Culture Collection, as well as other *Pseudomonas* sp. strains and three bacterial strains of other genera from China General Microbiological Culture Collection were tested. Bacteria in the log phase (100 μL) were mixed with 10 mL soft TSB agar (0.7% agar) and poured on top of a bottom layer containing 1.5% agar (15 mL). Then, the phage suspension was spotted onto the surface of double-layer agar plates containing lawns of the target bacterial strains. The plates were incubated at 25 °C for 12–18 h, and plaque formation was observed. Bacterial sensitivity to a phage was established based on the lysis-cleared zone around the spot. The results were classified into two categories: clear lysis zone and no lysis zone.

### 2.6. Stability of the Phage under Various Thermal, Ultraviolet, and pH Conditions

To evaluate the thermal stability of bacteriophages, phage preparations (10^10^ PFU/mL) in TSB broth were incubated in a 4 °C, 25 °C, 37 °C, or 50 °C water bath. To evaluate the ultraviolet (UV) stability of bacteriophages, phage preparations (10^10^ PFU/mL) in TSB broth were illuminated with a UV lamp (365 nm, 18 μW/cm^2^) for 0, 5, 15, 30, 45, or 60 min. Three samples were serially diluted. Subsequently, their titers were determined via the double-layer agar method, and the samples were placed in an incubator at 25 °C for 12–18 h. Three parallel experiments were conducted. To estimate pH stability, TSB was adjusted to various pH values (3.0, 4.0, 5.0, 6.0 7.0, 8.0, 9.0, 10.0, and 11.0). Next, 100 μL phage (10^10^ PFU/mL) was added to 900 μL TSB broth at each pH and incubated at 25 °C for 1 h.

### 2.7. In Vitro and In Vivo Phage Efficacy in Psa Control

To examine the lytic activity of PHB09 in vitro against a target bacterium, a bacteriophage aliquot was added to bacterial solution in the exponential phase (PsaBJ530 strain, 10^9^ CFU/mL) to achieve an MOI of 1. The solutions were mixed and incubated at 25 °C for 48 h with shaking (200 rpm). As the negative control, bacterial culture without phage was inoculated with the same volume of TSB medium. Bacterial growth was monitored using a TECAN microplate reader (TECAN, Männedorf, Switzerland). The OD_600_ was measured for 48 h. The lytic activity of PHB09 was determined based on the phage titer at the same time points. Three parallel tests were performed, and each experiment was conducted in triplicate.

In vivo experimentation to evaluate phage efficacy was performed with leaf discs (≈8 cm diameter). The discs were obtained from healthy kiwifruit plants. Leaf surface was disinfected with sodium hypochlorite 1% and washed twice with sterile distilled water. Subsequently, the leaf discs were placed in humidity chambers and inoculated individually with three drops of 100 µL of PsaBJ530 (10^9^ CFU/mL). In Psa + phage group, PHB09 was added two hours after Psa infection with a MOI of 1. Leaf discs were deposited in a plate containing 20 mL of sterile distilled water supplemented with cycloheximide (100 µg/mL) to avoid fungus growth [25]. Leaf samples from each group were collected at 0, 12, 24, 48 and 72 h after infection. For each sample, 0.5 cm^2^ leaf tissue was homogenized in 1 mL sterile TSB medium to determine Psa concentration (CFU/mL) and phage titer (PFU/mL). All experiments were performed in biological and technical triplicate.

### 2.8. DNA Extraction

The phage lysates were centrifuged at 8000× *g* for 5 min to remove cellular debris. The supernatant, containing the majority of viral particles, was filtered through a 0.22-μm syringe filter to remove cellular debris. The treated lysate was concentrated by centrifuging in a 100-kDa molecular weight cutoff ultrafiltration centrifuge tube (Amicon Ultra-15 centrifugal filter units; Millipore, MA, USA) at 5000× *g* to a final volume of 1 mL. The Nucleic Acid Extraction Kit II (Geneaid Biotech Ltd., Taiwan, China) was used to extract phage DNA from a high-titer plate lysate (minimum of 10^9^ PFU/mL). The extracted DNA was stored in a 1.5-mL ultracentrifuge tube at −20 °C until needed. DNA quality was evaluated by agarose gel electrophoresis. 

### 2.9. Genome Sequencing and Bioinformatics Analysis

Extracted PHB09 genomic DNA was sequenced on an Illumina sequencer (Illumina, San Diego, CA, USA). Raw reads were trimmed using Trimmomatic version 0.36 (parameters: version 0.36, illuminaclip: TruSeq3-PE.fa:2:30:10 leading:3 trailing:3 slidingwindow:4:15 minlen:40) [31] to obtain clean reads, which constituted more than 90% of the raw reads. Bowtie2 version 2.3.4 [32] was used to remove sequences of the host bacterial genome; high-quality clean reads were then assembled using IDBA-UD version 1.1.3 (parameters: kmer min 21, max 91, and Step 10) [33]. The final assemblies were filtered to obtain 2923 contigs. The filtered contigs were compared with clean reads using samtools v1.8. The GC content and average sequencing depth of the contigs were calculated. Contigs with avgDepth ≥ 100 and length ≥ 5 kb were selected, followed by the alignment to viral genomes with covering ≥50%, identity ≥80% by NCBI BLASTn (https://blast.ncbi.nlm.nih.gov/Blast.cgi, accessed on 20 October 2021). The circular viral genome was obtained according to the overlap of the two terminals of the sequence. The genes from assembled genomic sequences were predicted using GeneMarkS [34] (http://topaz.gatech.edu/GeneMark/genemarks.cgi, accessed 9 April 2021) and RAST [35] (http://rast.nmpdr.org/, accessed 9 April 2021). The program tRNAscan-SE was used to predict tRNA sequences [36]. The putative protein function associated with each open reading frame (ORF) was manually verified by searching the NCBI nonredundant protein sequence and conserved domain databases using the BLASTp, with the e-value to <1.0 × 10^−5^. The whole genome was compared with other nucleotide sequences using NCBI BLASTn (https://blast.ncbi.nlm.nih.gov/Blast.cgi, accessed on 20 October 2021). Subsequently, the average nucleotide identity was determined using the BLASTn alignment tool in the pyani package, and an interactive heatmap was constructed using heatmaply [37,38]. A genomic map was generated using CGview (http://cgview.ca/, accessed on 20 October 2021). The complete annotated genome sequence of phage PHB09, displayed in Table S1, was deposited in the NCBI nucleotide database under accession number OK040171. Easyfig v.2.0 (https://mjsull.github.io/Easyfig, accessed on 21 October 2021) was used to assess genomic organization and identify core genes in a given group of bacteriophages [39]. ViPTree (https://www.genome.jp/viptree, accessed on 6 November 2021) was used to generate viral proteomic trees for classification of viruses based on genome-wide similarities [40].

### 2.10. Phylogenetic Analysis

To elucidate the genome of PHB09, the large subunit of terminase and major capsid protein were compared phylogenetically with those of other bacteriophages (Table 1) using Mega-X software (version 10.1.6). ClustalX was used to align the inferred amino acid sequences using the default parameters. Based on the multiple-sequence alignment, the Jones–Taylor–Thornton model was employed for construction of a maximum likelihood tree with 100 bootstrap replicates.

### 2.11. Statistical Analysis 

Data analysis was performed using GraphPad Prism 8.0 (GraphPad Software, San Diego, CA, USA). Multiple t-tests were performed to determine the differences between groups at a significance level of *p* < 0.05.

## 3. Results and Discussion

### 3.1. Isolation and Morphology of Phage PHB09

**Figure 1:**
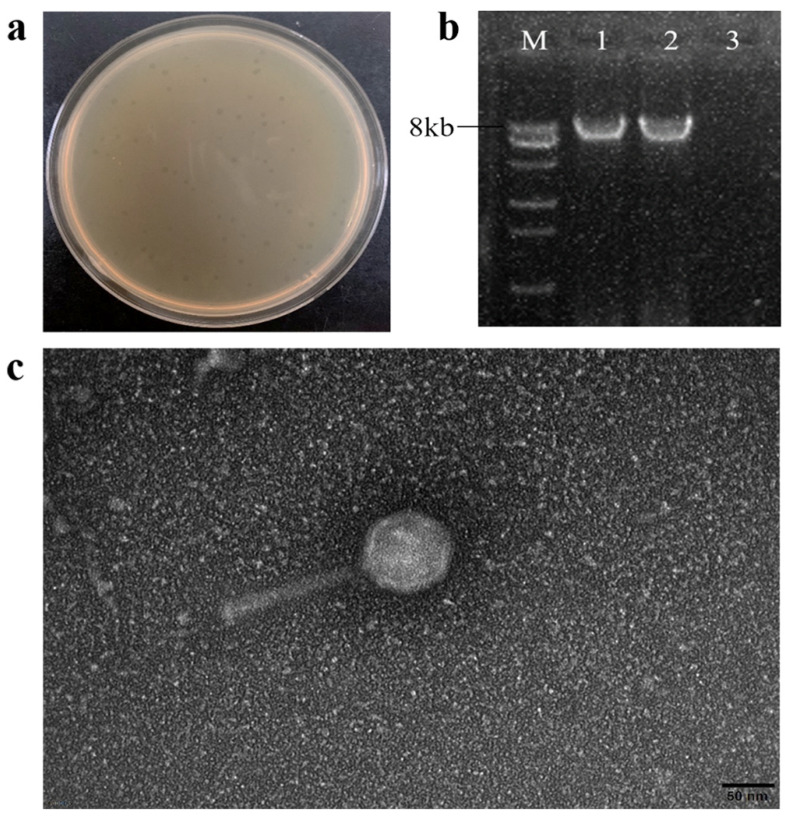
The morphology characteristics of phage PHB09. (**a**) Plaque morphology of phage PHB09. (**b**) Agarose gel electrophoresis of genomic segments. M: DNA marker, 1: PHB09 genome, 2: PHB09 genome digested with RNase, and 3: PHB09 genome digested with DNase. (**c**) Virion morphology observation of phage PHB09 under TEM.

A new *Pseudomonas* phage, vB_PsyM-PHB09 (designated phage PHB09 hereafter), was isolated from a kiwifruit orchard in Sichuan, China. The phage was purified by successive single-plaque isolations using the double-layer agar technique. Phage PHB09 formed clear plaques approximately 2 mm in diameter on a bacterial lawn of the host strain PsaBJ530 (Figure 1a). Enzyme digestion showed that PHB09 is a DNA phage, as it could be digested with DNase but not RNase (Figure 1b). Electron microscopy showed that PHB01 has an icosahedral head (height, 55.2 nm ± 1.0 nm; width, 54.5 nm ± 1.5 nm) and contractile tail (length, 145.0 nm ± 1.0 nm; width, 14.0 ± 0.5 nm) (Figure 1c).

Based on these morphological characteristics and according to the latest International Committee on Taxonomy of Viruses classification, PHB09 exhibited typical head and tail morphologies associated with the class *Caudoviricetes* (tailed ds-DNA phages). Viruses in all of these families have icosahedral or oblate heads but differ in the length and contractile abilities of their tails [41]. Electron microscopy showed that PHB09 was a myovirus [42]. Numerous studies have examined phages that infect *Pseudomonas* species, and more than 97% of those observed using electron microscopy belong to the *Caudovirales* [43]. Rombouts et al. tested a phage cocktail of five *Myoviridae* phages against 41 different strains of *P. syringae* pv. *porri* on leek leaves [44]. Frampton et al. characterized 24 Psa bacteriophages isolated from soil, water, and leaf samples collected in infected kiwifruit orchards, among which 22 belonged to *Myoviridae*, 1 to *Podoviridae*, and 1 to *Siphoviridae* [14]. Di Lallo et al. reported two new bacteriophages, phiPSA1 (*Siphoviridae*) and phiPSA2 (*Podoviridae*), which were isolated from kiwifruit leaves infected with Psa [45]. 

### 3.2. Biological Characterization of Phage PHB09 

**Figure 2:**
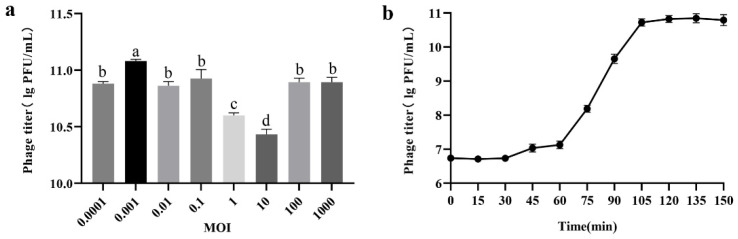
Biological characterization of phage PHB09. (**a**) Optimal multiplicity of infection (MOI) determination. (**b**) One-step growth curve. Values are means from three independent experiments. Means with the same letter are not significantly different from each other. Error bars indicate standard deviation.

Phage PHB09 generated a maximum titer of 1.91 × 10^11^ PFU/mL at an optimal MOI of 0.001 (Figure 2a). To determine the latent period and burst size of phage PHB09, a one-step growth curve was plotted at 25 °C. The latent period was approximately 60 min and the rise period approximately 40 min (Figure 2b). Furthermore, the burst size of phage PHB09 was estimated at 182 PFU/infected cell, indicating that phage PHB09 replicates efficiently in Psa but requires a long latency period.

### 3.3. Thermal, Ultraviolet, and pH Stability Tests

**Figure 3:**
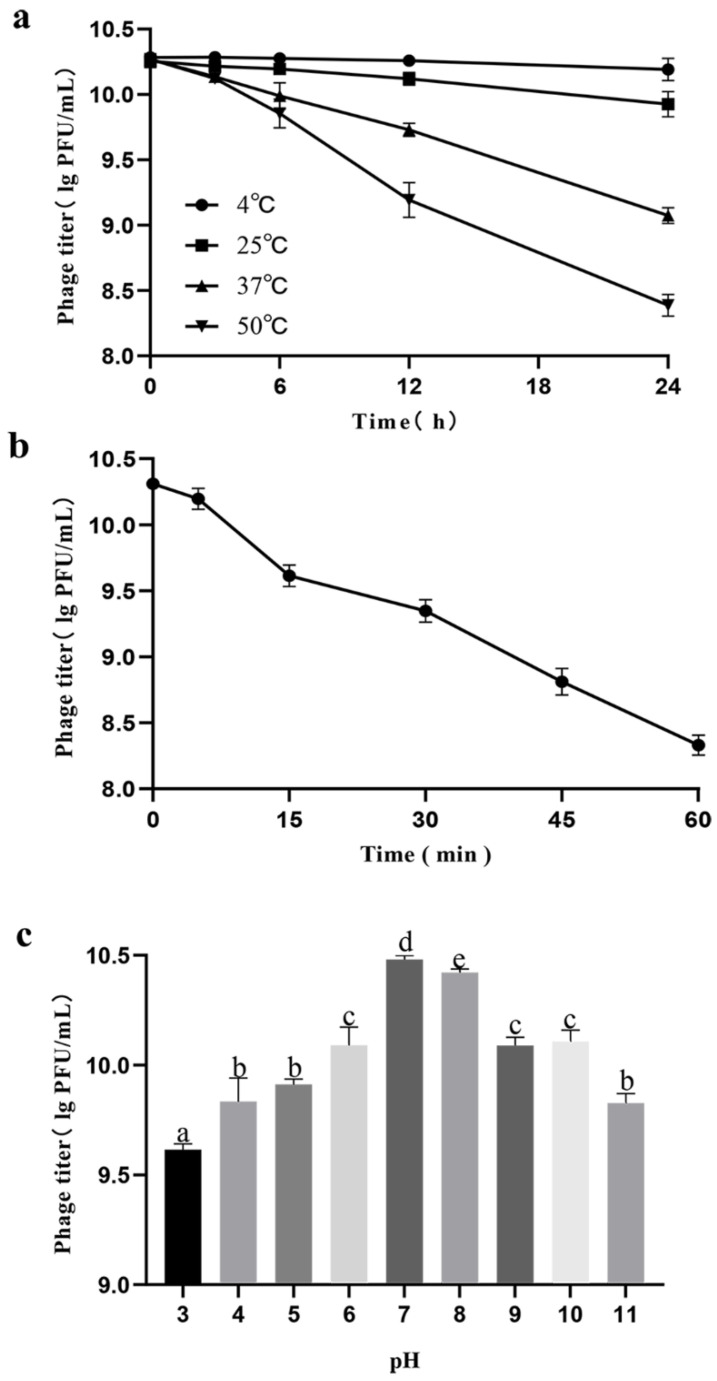
Stability of phage PHB09 in different conditions. (**a**) Stability at different temperature. (**b**) Stability to exposure of ultraviolet light. (**c**) Stability to changes in pH value. Values are means from three independent experiments. Means with the same letter are not significantly different from each other. Error bars indicate standard deviation.

The stability of lytic activity against target bacteria is important for phage therapy. Thermal stability tests showed that PHB09 activity was high following water incubation from 4 °C to 37 °C for 12 h, after which it began to decrease. However, the maximum decrease was only 1.47 log PFU/mL after 24 h when the samples were held at a temperature of 37 °C. When the temperature was increased to 50 °C, the phage titer decreased by 2.08 log PFU/mL after 24 h of incubation. Compared with the samples at 4 °C and 25 °C, phage titers decreased significantly at 37 °C and 50 °C after 6 h treatment (Figure 3a). UV stability tests showed that PHB09 retained high levels of activity from 0 to 60 min treatment. With increasing UV exposure time, the phage titer gradually decreased. When phage PHB09 was exposed to UV for 60 min, a titer reduction of 2.13 log PFU/mL was observed (Figure 3b). To evaluate pH stability, the phage was treated with acidic, neutral, and basic TSB medium for 1 h. PHB09 remained stable over a pH range of 3–11. However, it showed significant reductions at pH 3 and 11, with titer decreases of 0.97 and 0.88 log PFU/mL, respectively (Figure 3c).

Environmental factors exert significant effects on phage stability, impeding the efficacy of phage treatment. For bacteriophages to be used as biocontrol agents, their lytic activity must be stable under various environmental conditions such as temperature, UV light, and soil pH [16]. Temperature plays a fundamental role in the attachment, penetration, and amplification of phage particles within their host cells [46]. At low temperatures, genetic material from only a few phages enters bacterial host cells, and therefore fewer phage particles are involved in the multiplication phase. On the other hand, high temperatures promote an extended phage latency period. In our study, Phage PHB09 was stable over a wide range of temperatures from 4 °C to 37 °C, which is a significant advantage for future applications. Thus, in summer, when temperatures occasionally rise to 37 °C, the phage maintains its activity. As the most critical period for plants is from autumn through winter to early spring, and the infective ability is reduced at temperatures above 25 °C [47], temperature is not a problem for implementation of this phage in therapy. Solar radiation or UV irradiation can directly affect free viruses via degradation of proteins, alteration of the viral structure, and reduction of infectivity [48]. The abundance of phage PHB09 particles decreased when exposed to UV radiation. However, sensitivity to UV wavelengths in solar radiation can be overcome by applying the phage at high titers and late in the day or at night, a period during which radiation is limited [49]. pH is another important factor affecting phage attachment, infectivity, intracellular replication, and amplification [50]. Unfavorable pH values can interfere with the lysozyme enzyme or with other phage capsid proteins, thereby preventing phage attachment to receptor sites on the host cell. Phage PHB09 exhibited high pH tolerance, as indicated by maintenance of a stable titer across a pH range of 3–11. In addition, kiwifruit grow optimally at pH 5.5–6.0, and the temperature at which kiwifruit canker grows in Sichuan Province is below 30 °C. Based on these environmental conditions, PHB09 bacteriophages are likely to be stable in the natural growth environment of kiwifruit.

### 3.4. Host Range of Phage PHB09

The host range of phage PHB09 was investigated by infecting 22 bacterial strains of different genera and species using the spot assay method. The results, shown in Table 2, indicated that phage PHB09 could lyse not only biovar 3 Psa strains BJ530, BJ9, and BST isolated in the laboratory but also other biovar 2 Psa strains from the Korean Agriculture Culture Collection that are considered highly virulent plant pathogens and causative agents of kiwifruit losses worldwide. Other tested *Pseudomonas* sp. strains were not infected.

Lytic phages can kill the host bacterium and play an important role in maintaining biodiversity [14]. With advantages such as target specificity and rapid self-replication, phages are being explored as an alternative strategy for the treatment of *Pseudomonas* infections [51]. In the host range experiment, PHB09 showed a narrow host range, with a limited ability to infect Psa and an inability to infect other *Pseudomonas* sp. strains. This result indicates that PHB09 is a promising biocontrol agent that can specifically inactivate Psa strains. 

### 3.5. In Vitro and In Vivo Efficacy of Phage PHB09 

**Figure 4:**
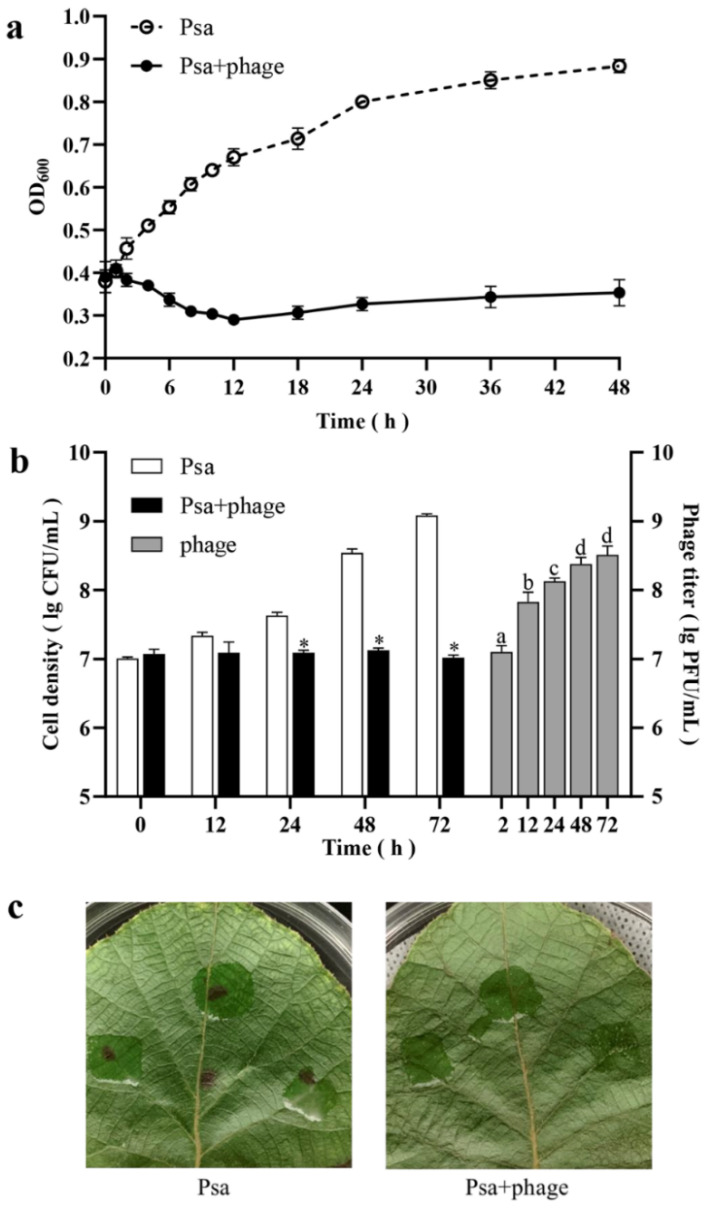
Efficacy of Phage PHB09 against Psa. (**a**) Results of in vitro experiment. Cell density are shown by OD_600_ at different time points. (**b**) Results of in vivo experiment. White and black bars represent cell density (CFU/mL) of two treatment groups, gray bars represent the phage titer (PFU/mL) in Psa + phage group. (**c**) The kiwifruit leaves of two groups 72 h after Psa inception. Values are means from three independent experiments. * Indicates significant difference between Psa and Psa + phage groups (*p* < 0.05). Means with the same letter are not significantly different from each other. Error bars indicate standard deviation.

To further assess the application potential of phage PHB09, the phage was used to inhibit the growth of Psa in vitro. To examine the duration of PHB09 lytic activity against the target bacterium in vitro, the cell density (OD_600_) was measured over 48 h (Figure 4a). The density of PsaBJ530 without phage increased by 0.49 after 48 h. The density of bacteria treated with the phage decreased gradually, to 0.29 at 12 h, and then slowly increased until 48 h. After 48 h of phage treatment, the bacterial concentration was significantly lower than that in non-treated cultures.

To test the efficacy of phage PHB09 against bacterial canker in vivo, kiwifruit leaves were treated by PHB09 two hours after Psa infection. The results showed that PHB09 could reduce the Psa load over kiwifruit leaves 24–72 h post-infection (Figure 4b). Phage PHB09 was detectable throughout the entire experiment, showing a significant titer increase at 12 h (Figure 4b). After 72 h, leaf infected with Psa had typical symptoms (browning and cankering), while leaf infected with Psa and treated with PHB09 showed no symptoms (Figure 4c). These results indicate that the treatment of phage PHB09 can efficiently control bacterial canker in kiwifruit.

Based on these results, PHB09 can maintain stable lytic activity against PsaBJ530 both in vitro and in vivo, highlighting the potential of PHB09 in the biological control of Psa infection. The lytic effect pattern is very similar to those in previous reports of Psa phages (KHUφ34, KHUφ38, and PPPL-1) [11,28]. This similarity indicates that PHB09 can be considered a promising candidate for phage therapy, and that its characterization in this study may provide a starting point for further exploration of its potential in the biological control of bacterial canker of kiwifruit. Due to the systemic nature of bacterial canker of kiwifruit [52], phage therapy is best used as a preventative strategy. As phage particles can move in moist environments such as plant tissues in addition to infecting bacteria that are on the plant’s surface, they can be used to assess and control bacteria within a plant’s tissues during an infection. However, the natural environment includes water, wind, rain, and sunlight, which influence the success of phage treatment, and no studies to date have reported the effects of these factors. In the future, protective formulations and carrier bacteria [53] must be identified to improve viral survival. Further studies are needed for this purpose. 

### 3.6. Genome Characterization and Comparative Genomic Analysis 

**Figure 5:**
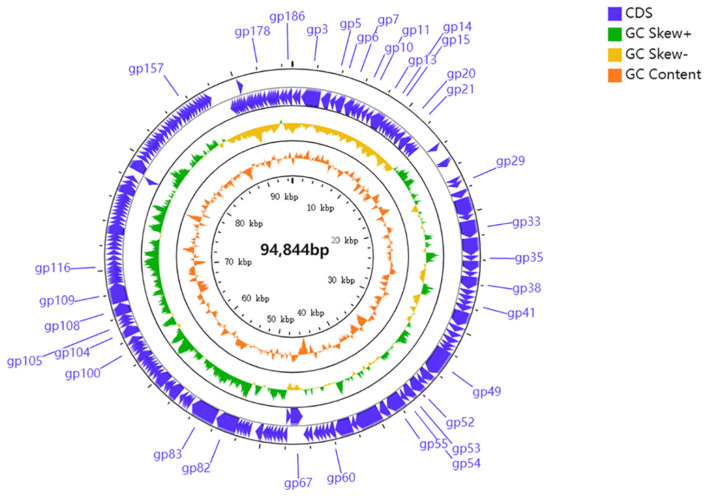
Genomic map of PHB09. Circles from outermost to innermost correspond to predicted genes (BLASTp, nr database, E value of <0.00001) on forward strand; reverse strand; GC content.

The 94,844-bp genome of PHB09 is circular, with a GC content of 57.61% (accession number: OK040171). In total, 186 genes were predicted, and no tRNA genes were identified. The majority of the predicted genes were detected on the forward strand, accounting for 74.2% of the total (138 of 186). Thirty-four genes had significant similarity to genes with known functions, while over 80% of all predicted genes were hypothetical proteins that could not be annotated to any homologs (BLASTp; e-value cutoff 10^−5^). Based on the results of BLASTp and Conserved Domain Database analyses, 34 genes were annotated to encode functional proteins (Table 3), and their arrangement at the whole-genome level was mapped (Figure 5). Although the complete genome of phage PHB09 has been analyzed, many of its proteins have unknown functions. Genome sequencing suggested that no previously described antibiotic resistance or virulence factor genes are present in the genome of phage PHB09; this characteristic increases the safety of this phage for agricultural application (Table S1). 

**Figure 6:**
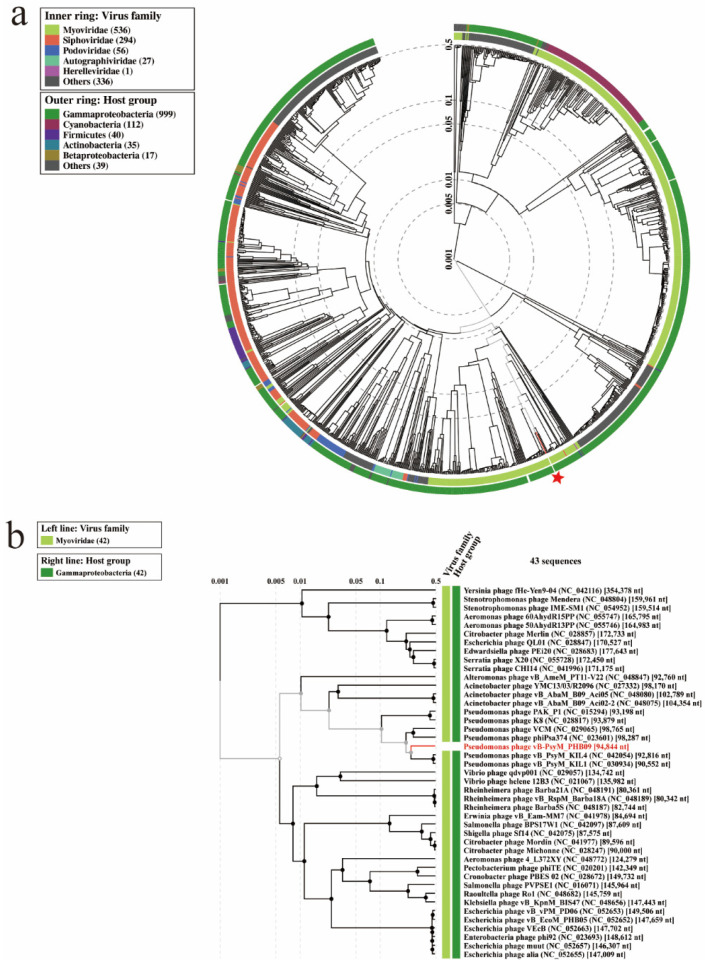
Proteomic relationships between PHB09 and other phages. (**a**) The dendrogram represents proteome of PHB09 with 4240 prokaryotic dsDNA virus genomes in wide similarity relationships. The red-colored star mark indicates phage PHB09. (**b**) A proteomic tree base on tBLASTx genomic sequence comparisons of 43 bacteriophage genomes.

**Figure 7:**
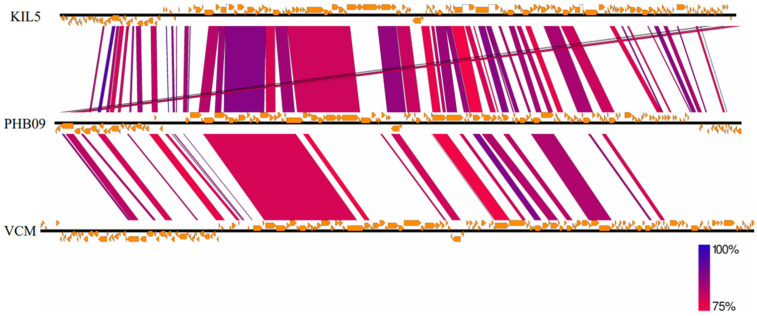
The genome sequence similarity between phage VCM, vB_PsyM_KIL5, and PHB09. The figure was generated via Easyfig v.2.0. Predicted genes and the direction of transcription are indicated by frames. Conserved regions are shaded, with the color intensity indicating the nucleotide sequence identity level (from 75 to 100%).

**Figure 8:**
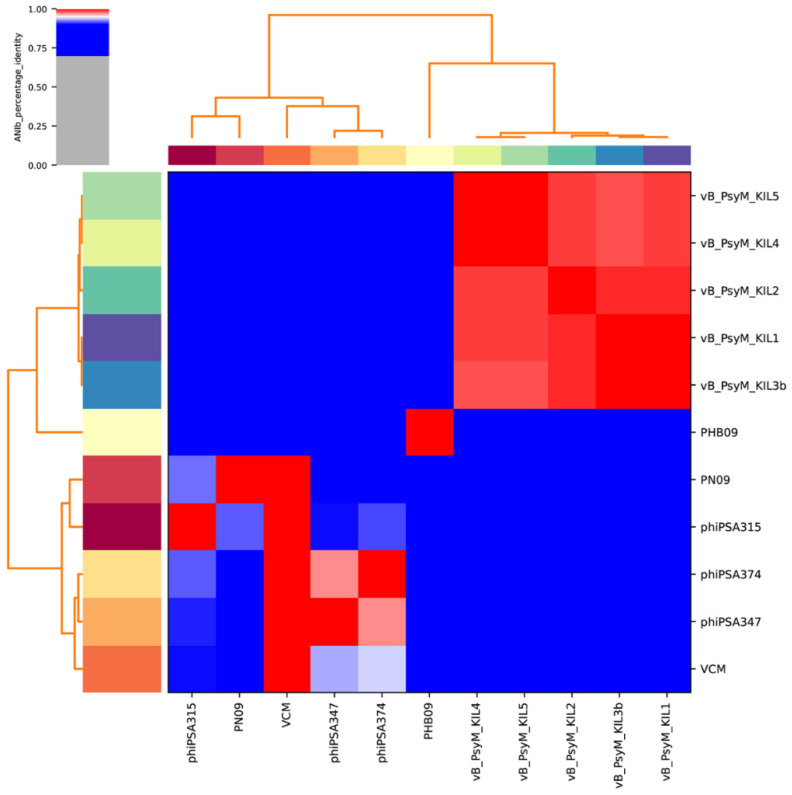
Heatmap of 11 whole genomes. Sequences were from related *Otagovirus* phages and *Flaumdravirus* phages in the NCBI database (https://blast.ncbi.nlm.nih.gov/Blast.cgi, accessed on 20 October 2021). The average nucleotide identity (ANI) was valued. Values range from 0 (0%) ANI to 1 (100% ANI): gray represents 0% ANI; clusters of highly similar phages are highlighted in pink and red.

Proteomic tree of PHB09 was generated based on genomic similarity scores (denoted by SG) derived from tBLASTx scores (Figure 6a). The viral proteomic tree revealed that phage PHB09 has a high sequence identity to *Pseudomonas* phage VCM, belonging to genus *Otagovirus* (query coverage 37%; identity 78.75%; accession number LN887844.1), and to *Pseudomonas* phage vB_PsyM_KIL5, belonging to genus *Flaumdravirus* (query coverage 51%; identity 88.29%; accession number KU130130.1) (Figure 6b). The whole genome of PHB09 was colinear with those two Psa phage genomes, with low similarity in terms of genomic architecture (Figure 7). The heatmap results were similar to the NCBI search results based on the complete genome of phage PHB09, indicating that phage PHB09 has low genomic similarity to known phages (Figure 8). 

The Bacterial and Archaeal Viruses Subcommittee [54] defines a genus as a cohesive group of viruses sharing a high degree of nucleotide identity (>50%) and being distinct from viruses in other genera. Thus, phage PHB09 might be classified as belonging to a novel genus.

### 3.7. Phylogenetic Analysis

**Figure 9:**
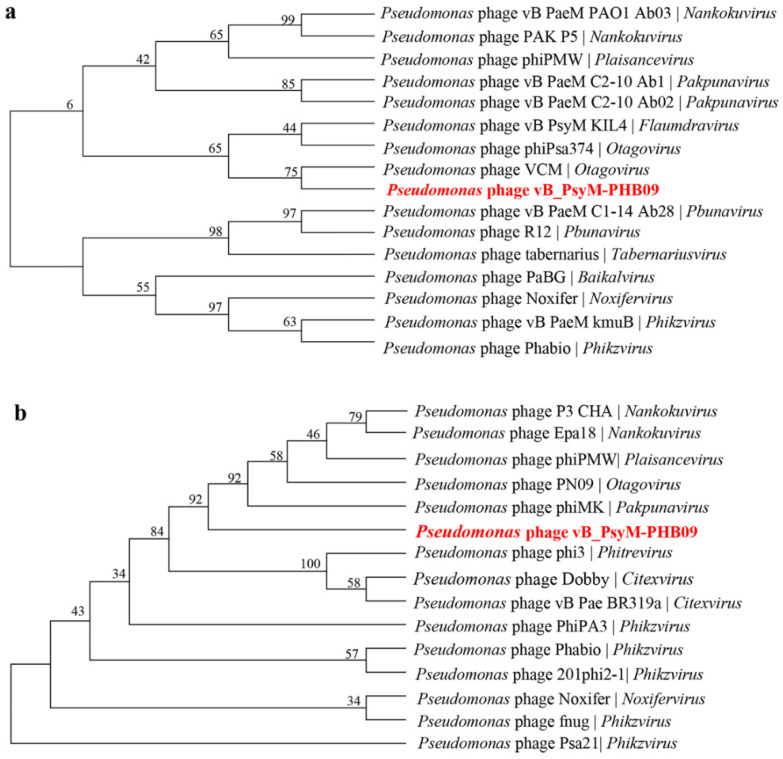
Phylogenetic relationship of phage PHB09. (**a**) Phylogenetic tree based on the amino acid sequences of the terminase large subunits. (**b**) Phylogenetic tree based on the amino acid sequences of major capsid proteins.

Two phylogenetic trees were constructed using the amino acid sequences of the predicted large subunit of terminase and the major capsid protein, which are often the most conserved sequences in phage genomes. Phage PHB09 is highly homologous to *Pseudomonas* phage VCM based on the terminase large subunit, and this sequence was distinct from those of other groups (Figure 9a). Phylogenetic analysis using the sequences of the major capsid protein placed PHB09 on a separate branch, displaying a distant phylogenetic relationship to the *Otagovirus* genus (Figure 9b). According to phylogenetic analysis of the complete genome sequence and amino acid sequences of conserved proteins (major capsid protein and terminase large subunit), phage PHB09 belongs to a new phage lineage, indicating that PHB09 is a novel genus in the class *Caudoviricete* [55]. 

## 4. Conclusions

In conclusion, this study describes the isolation, characterization, and application of vB_PsyM-PHB09, a novel DNA phage against Psa. It exhibits a short latent period, large burst size, and stability across a broad range of pH and temperature conditions. Both morphological and genetic analyses indicated that PHB09 is a member of class *Caudoviricete*. However, PHB09 does not belong to any genera previously identified in myovirus and should be assigned to a new genus. This study of PHB09 enriches the research on *Pseudomonas* phages. Moreover, PHB09 exhibited a strong ability to kill Psa strains both in vitro and in vivo, suggesting the potential for biocontrol of Psa in kiwifruit agriculture. 

## Figures and Tables

**Table 1:** Accession numbers of genes used in phylogenetic analysis.

Accession No.	Protein	Organism	Genus
HE983845	Terminase large subunit	*Pseudomonas* phage vB_PaeM_C2-10_Ab1	*Pakpunavirus*
CEF88981	Terminase large subunit	*Pseudomonas* phage vB_PaeM_C2-10_Ab02	*Pakpunavirus*
YP_009616728	Terminase large subunit	*Pseudomonas* phage vB_PsyM_KIL4	*Flaumdravirus*
NC_041994	Terminase large subunit	*Pseudomonas* phage Noxifer	*Noxifervirus*
YP_009124453	Terminase large subunit	*Pseudomonas* phage vB_PaeM_PAO1_Ab03	*Nankokuvirus*
YP_008857039	Terminase large subunit	*Pseudomonas* phage PAK_P5	*Nankokuvirus*
YP_008433440	Terminase large subunit	*Pseudomonas* phage PaBG	*Baikalvirus*
QPB10483	Terminase large subunit	*Pseudomonas* phage VCM	*Otagovirus*
QNO00383	Terminase large subunit	*Pseudomonas* phage phiPsa374	*Otagovirus*
YP_009966987	Terminase large subunit	*Pseudomonas* phage vB_PaeM_C1-14_Ab28	*Pbunavirus*
YP_009914209	Terminase large subunit	*Pseudomonas* phage R12	*Pbunavirus*
YP_001956731	Terminase large subunit	*Pseudomonas* phage vB_PaeM_kmuB	*Phikzvirus*
ARV76634	Terminase large subunit	*Pseudomonas* phage Phabio	*Phikzvirus*
YP_009595351	Terminase large subunit	*Pseudomonas* phage phiPMW	*Plaisancevirus*
YP_009620857	Terminase large subunit	*Pseudomonas* phage tabernarius	*Tabernariusvirus*
YP_009291185	Major capsid protein	*Pseudomonas* phage phiMK	*Pakpunavirus*
ALY08259	Major capsid protein	*Pseudomonas* phage phi3	*Phitrevirus*
YP_009595355	Major capsid protein	*Pseudomonas* phage phiPMW	*Plaisancevirus*
ADX32002	Major capsid protein	*Pseudomonas* phage P3_CHA	*Nankokuvirus*
YP_009609037	Major capsid protein	*Pseudomonas* phage Noxifer	*Noxifervirus*
AZF87846	Major capsid protein	*Pseudomonas* phage Dobby	*Citexvirus*
QJB22791	Major capsid protein	*Pseudomonas* phage fnug	*Phikzvirus*
QVJ13065	Major capsid protein	*Pseudomonas* phage Psa21	*Phikzvirus*
AEH03559	Major capsid protein	*Pseudomonas* phage PhiPA3	*Phikzvirus*
ARV76818	Major capsid protein	*Pseudomonas* phage Phabio	*Phikzvirus*
QPB10488	Major capsid protein	*Pseudomonas* phage PN09	*Otagovirus*
YP_001956923	Major capsid protein	*Pseudomonas* phage 201phi2-1	*Phikzvirus*

**Table 2:** Host range of phage vB_PsyM-PHB09.

Bacteria Strain	Accession Number	Source	Plaque Formation ^a^
Psa BJ530 (biovar 3)	1.19157	CGMCC	+
Psa BJ9 (biovar 3)		Isolate from kiwi orchard	+
Psa BST (biovar 3)		Isolate from kiwi orchard	+
Psa (biovar 2)	10584	KACC	+
Psa (biovar 2)	10592	KACC	+
Psa (biovar 2)	10587	KACC	+
*Pseudomonas oryzihabitans*	1.3158	CGMCC	−
*Pseudomonas oryzihabitans*	1.2879	CGMCC	−
*Pseudomonas oryzihabitans*	1.15148	CGMCC	−
*Pseudomonas fluorescens*	1.7375	CGMCC	−
*Pseudomonas fluorescens*	1.7373	CGMCC	−
*Pseudomonas fluorescens*	1.7306	CGMCC	−
*Pseudomonas putida*	1.8829	CGMCC	−
*Pseudomonas putida*	1.7662	CGMCC	−
*Pseudomonas gessardii*	1.6429	CGMCC	−
*Pseudomonas gessardii*	1.874	CGMCC	−
*Pseudomonas fragi*	1.7759	CGMCC	−
*Pseudomonas fragi*	1.7757	CGMCC	−
*Pseudomonas fragi*	1.7752	CGMCC	−
*Enterobacter hormaechei*	1.10608	CGMCC	−
*Enterobacter cloacae*	1.8726	CGMCC	−
*Escherichia coli*	1.12883	CGMCC	−

^a^ +, susceptible to PHB09; −, unsusceptible to PHB09. KACC: Korean Agricultural Culture Collection. CGMCC: China General Microbiological Culture Collection Center.

**Table 3:** Gene similarities of PHB09.

Gene Product	Genebank ID	Identity (%)	e-Value	Putative Function	Organism	Functional Categories
Gp3	UAV89895.1	81.28	0	nicotinamide phosphoribosyltransferase	*Pseudomonas* phage REC	auxiliary metabolism
Gp5	UAV89899.1	90	3.53 × 10^−84^	polynucleotide kinase	*Pseudomonas* phage REC	nucleotides metabolism
Gp6	QNO00057.1	76.28	6.22 × 10^−163^	RNA ligase	*Pseudomonas* phage phiPsa300	transcription
Gp7	WP_198844968.1	71.79	6.1 × 10^−139^	SPFH domain-containing protein	*Pseudomonas* sp. MF7453	structure
Gp10	UAV89634.1	87.30	2.25 × 10^−123^	phosphohydrolase	*Pseudomonas* phage M5.1	nucleotides metabolism
Gp11	UAV89635.1	65.60	5.35 × 10^−70^	hydrolase	*Pseudomonas* phage M5.1	auxiliary metabolism
Gp13	AMR57593.1	79.14	0	putative DNA ligase	*Pseudomonas* phage vB_PsyM_KIL3	DNA replication and repair
Gp14	ATN92916.1	55.12	1.17 × 10^−17^	putative DNA ligase	*Pseudomonas* phage PPSC2	DNA replication and repair
Gp15	YP_009616712.1	81.59	9.59 × 10^−98^	putative deoxycytidylate deaminase	*Pseudomonas* phage vB_PsyM_KIL4	auxiliary metabolism
Gp20	AMR57443.1	95.61	1.5 × 10^−142^	putative serine protease	*Pseudomonas* phage vB_PsyM_KIL2	auxiliary metabolism
Gp21	AMR57444.1	97.67	0	putative phosphate starvation protein	*Pseudomonas* phage vB_PsyM_KIL2	nucleotides metabolism
Gp29	AMR57452.1	83.33	3.56 × 10^−81^	putative HNH endonuclease	*Pseudomonas* phage vB_PsyM_KIL2	DNA replication and repair
Gp33	YP_009222712.1	94.27	0	terminase-like family protein	*Pseudomonas* phage VCM	packing
Gp35	YP_009275981.1	91.94	6.26 × 10^−95^	putative methyltransferase	*Pseudomonas* phage vB_PsyM_KIL1	nucleotides metabolism
Gp38	YP_009616734.1	97.73	0	major capsid protein	*Pseudomonas* phage vB_PsyM_KIL4	structure
Gp41	UAV89665.1	81.96	9.54 × 10^−70^	head-to-tail stopper	*Pseudomonas* phage M5.1	structure
Gp49	YP_009616750.1	85.96	0	putative tape measure protein	*Pseudomonas* phage vB_PsyM_KIL4	structure
Gp52	YP_009276001.1	93.48	0	putative structural protein	*Pseudomonas* phage vB_PsyM_KIL1	structure
Gp53	YP_009276002.1	88.53	9.3 × 10^−169^	putative baseplate protein	*Pseudomonas* phage vB_PsyM_KIL1	structure
Gp54	YP_009276003.1	90.08	8.85 × 10^−76^	putative tail lysozyme	*Pseudomonas* phage vB_PsyM_KIL1	lysis
Gp55	YP_009276004.1	91.34	0	putative baseplate component	*Pseudomonas* phage vB_PsyM_KIL1	structure
Gp60	YP_009276009.1	94.62	5.6 × 10^−129^	putative endolysin	*Pseudomonas* phage vB_PsyM_KIL1	lysis
Gp67	AMR57493.1	85.26	0	putative RNA ligase	*Pseudomonas* phage vB_PsyM_KIL2	transcription
Gp82	YP_009276030.1	93.82	0	putative DNA primase/helicase	*Pseudomonas* phage vB_PsyM_KIL1	DNA replication and repair
Gp83	UAV89975.1	80.31	0	DNA polymerase	*Pseudomonas* phage REC	DNA replication and repair
Gp100	YP_009276002.1	88.53	9.3 × 10^−169^	putative baseplate protein	*Pseudomonas* phage vB_PsyM_KIL1	structure
Gp104	UAV89994.1	76.03	1.3 × 10^−173^	flavin-dependent thymidylate synthase	*Pseudomonas* phage REC	nucleotides metabolism
Gp105	UAV84600.1	100	4.3 × 10^−105^	HNH homing endonuclease	*Pseudomonas* phage PHB09	auxiliary metabolism
Gp108	YP_009222647.1	90.46	0	ribonucleotide-diphosphate reductase subunit beta	*Pseudomonas* phage VCM	DNA replication and repair
Gp109	YP_009222646.1	95.48	0	ribonucleoside-diphosphate reductase NrdZ	*Pseudomonas* phage VCM	nucleotides metabolism
Gp116	YP_009276062.1	87.83	2.22 × 10^−40^	putative glutaredoxin	*Pseudomonas* phage vB_PsyM_KIL1	nucleotides metabolism
Gp157	AXF53051.1	43.37	2.94 × 10^−15^	NADH oxidase H2O-forming	*Siphoviridae* sp.	auxiliary metabolism
Gp178	UAV89606.1	74.21	4.59 × 10^−74^	peptide chain release factor	*Pseudomonas* phage M5.1	auxiliary metabolism
Gp186	YP_009222746.1	82.19	3.4 × 10^−114^	DNA recombination-mediator protein A	*Pseudomonas* phage VCM	DNA replication and repair

## Data Availability

The complete genome sequence of phage vB_PsyM-PHB09 was submitted to the GenBank database under accession number OK040171.

## Supplementary Materials

The following are available online at https://www.mdpi.com/article/10.3390/v13112275/s1, Table S1: title, the annotation of phage PHB09 genome.
