# Supplementary material for: Isolation of the Novel Phage PHB09 and Its Potential Use against the Plant Pathogen Pseudomonas syringae pv. actinidiae
Source: Viruses. 2021 Nov 14;13(11):2275. doi: 10.3390/v13112275 (PMC8622976; doi:10.3390/v13112275)
Supplement: Supplementary file 1 [file viruses-13-02275-s001.zip › viruses-1436966-supplementary.pdf]

Table S1. The annotation of phage PHB09 genome.

Gene	Start	End	Length	Strand	Predicted function of the gene product	e-value	Identity(%)	Organism
1	274	131	144	-	hypothetical protein PJG4_015	4.48e-06	59.091	Pseudomonas phage JG004
2	755	255	501	-	hypothetical protein PPSC2_140	8.95e-61	61.585	Pseudomonas phage PPSC2
3	2592	799	1794	-	nicotinate phosphoribosyltransferase	0	81.282	Pseudomonas phage REC
4	3523	2693	831	-	hypothetical protein PN09_032	3.88e-65	44.231	Pseudomonas phage PN09
5	3983	3591	393	-	polynucleotide kinase	3.53e-84	90	Pseudomonas phage REC
6	4873	3980	894	-	RNA ligase	6.22e-163	76.289	Pseudomonas phage phiPsa300
7	5747	4923	825	-	SPFH domain-containing protein	6.06e-139	71.795	Pseudomonas sp. MF7453
8	5938	5744	195	-	hypothetical protein PN09_032	1.54e-41	93.151	Pseudomonas phage PN09
9	6160	5939	222	-	hypothetical protein BH774_gp017	1.54e-41	93.151	Pseudomonas phage vB_PsyM_KIL1
10	6729	6160	570	-	phosphoesterase	2.25e-123	87.302	Pseudomonas phage M5.1
11	7201	6722	480	-	hydrolase	5.35e-70	65.605	Pseudomonas phage M5.1
12	7666	7259	408	-	hypothetical protein vB_PsyM_KIL5_0021	2.17e-91	95.556	Pseudomonas phage vB_PsyM_KIL5
13	8840	7677	1164	-	putative DNA ligase	0	79.146	Pseudomonas phage vB_PsyM_KIL3
14	9070	8837	234	-	putative DNA ligase	1.17e-17	55.128	Pseudomonas phage PPSC2
15	9561	9067	495	-	putative deoxycytidylate deaminase	9.59e-98	81.595	Pseudomonas phage vB_PsyM_KIL4
16	9721	9551	171	-	hypothetical protein PN09_045	2.80e-13	61.818	Pseudomonas phage PN09
17	9945	9721	225	-	hypothetical protein M51_41	1.38e-31	70.27	Pseudomonas phage M5.1
18	10265	9996	270	-	hypothetical protein PPSC2_159	1.35e-59	100	Pseudomonas phage PPSC2
19	10488	10258	231	-	hypothetical protein PPSC2_160	1.08e-28	73.684	Pseudomonas phage PPSC2

20	11168	10554	615	-	putative serine protease	1.52e-142	95.61	Pseudomonas phage vB_PsyM_KIL2
21	11937	11161	777	-	putative phosphate starvation protein	0	97.674	Pseudomonas phage vB_PsyM_KIL2
22	12332	11979	354	-	hypothetical protein vB_PsyM_KIL2_0034	2.89e-67	86.325	Pseudomonas phage vB_PsyM_KIL2
23	12531	12334	198	-	hypothetical protein PPSC2_164	1.66e-06	41.667	Pseudomonas phage PPSC2
24	12959	12528	432	-	hypothetical protein FDI83_gp034	1.11e-68	80.451	Pseudomonas phage vB_PsyM_KIL4
25	13784	14101	318	+	hypothetical protein REC_48	6.40e-64	94.286	Pseudomonas phage REC
26	15324	15446	123	+	hypothetical protein			
27	15483	15608	126	+	hypothetical protein BH774_gp158	9.49e-20	97.561	Pseudomonas phage vB_PsyM_KIL1
28	17067	17201	135	+	hypothetical protein			
29	17297	17713	417	+	putative HNH endonuclease	3.56e-81	83.333	Pseudomonas phage vB_PsyM_KIL2
30	18064	18687	624	+	hypothetical protein BH774_gp155	7.42e-63	44	Pseudomonas phage vB_PsyM_KIL1
31	18723	20273	1551	+	hypothetical protein KKIINIFJ_00071	1.20e-85	42.361	Pseudomonas phage PN05
32	20290	20511	222	+	hypothetical protein psageK4_063	1.75e-16	63.014	Pseudomonas phage psageK4
33	20659	22128	1470	+	terminase-like family protein	0	94.274	Pseudomonas phage VCM
34	22137	23606	1470	+	hypothetical protein BH774_gp151	0	98.364	Pseudomonas phage vB_PsyM_KIL1
35	23615	24064	450	+	putative methyltransferase	6.26e-95	91.946	Pseudomonas phage vB_PsyM_KIL1

36	24061	24975	915	+	hypothetical protein BH774_gp149	0	96.053	Pseudomonas phage vB_PsyM_KIL1
37	25000	25386	387	+	hypothetical protein BH774_gp148	4.48e-82	96.094	Pseudomonas phage vB_PsyM_KIL1
38	25400	26461	1062	+	major capsid protein	0	97.734	Pseudomonas phage vB_PsyM_KIL4
39	26517	26999	483	+	hypothetical protein BH774_gp146	1.96e-105	91.25	Pseudomonas phage vB_PsyM_KIL1
40	26983	27450	468	+	hypothetical protein BH774_gp145	1.36e-103	94.156	Pseudomonas phage vB_PsyM_KIL1
41	27461	27829	369	+	head-to-tail stopper	9.54e-70	81.967	Pseudomonas phage M5.1
42	27826	28395	570	+	hypothetical protein vB_PsyM_KIL2_0064	8.72e-127	89.947	Pseudomonas phage vB_PsyM_KIL2
43	28407	29690	1284	+	hypothetical protein VCM_00104	0	84.543	Pseudomonas phage VCM
44	29723	30247	525	+	hypothetical protein BH774_gp139	1.44e-121	98.851	Pseudomonas phage vB_PsyM_KIL1
45	30311	30814	504	+	hypothetical protein BH774_gp138	5.8e-106	89.222	Pseudomonas phage vB_PsyM_KIL1
46	30825	31358	534	+	hypothetical protein BH774_gp137	2.12e-117	93.22	Pseudomonas phage vB_PsyM_KIL1
47	31372	31734	363	+	hypothetical protein BH774_gp135	2.85e-73	93.333	Pseudomonas phage vB_PsyM_KIL1
48	31758	31985	228	+	hypothetical protein	2.27e-41	86.667	Pseudomonas phage vB_PsyM_KIL4
49	31996	34344	2349	+	putative tape measure protein	0	85.969	Pseudomonas phage vB_PsyM_KIL4

50	34344	35108	765	+	hypothetical protein BH774_gp132	1.89e-166	87.795	Pseudomonas phage vB_PsyM_KIL1
51	35118	35459	342	+	hypothetical protein BH774_gp131	2.20e-75	95.575	Pseudomonas phage vB_PsyM_KIL1
52	35465	36388	924	+	putative structural protein	0	93.485	Pseudomonas phage vB_PsyM_KIL1
53	36385	37149	765	+	putative baseplate protein	9.3e-169	88.538	Pseudomonas phage vB_PsyM_KIL1
54	37151	37516	366	+	putative tail lysozyme	8.85e-76	90.083	Pseudomonas phage vB_PsyM_KIL1
55	37520	38977	1458	+	putative baseplate component	0	91.34	Pseudomonas phage vB_PsyM_KIL1
56	38990	39724	735	+	hypothetical protein FDI83_gp133	2.2e-157	90.574	Pseudomonas phage vB_PsyM_KIL4
57	39734	42010	2277	+	hypothetical protein VCM_00091	3.4e-178	42.699	Pseudomonas phage VCM
58	42010	42288	279	+	hypothetical protein	1.43e-09	30.864	Pseudomonas flexibilis
59	42334	43809	1476	+	hypothetical protein VCM_00089	0	63.211	Pseudomonas phage VCM
60	43809	44369	561	+	putative endolysin	5.6e-129	94.624	Pseudomonas phage vB_PsyM_KIL1
61	44380	44631	252	+	hypothetical protein BH774_gp121	7.02e-51	97.59	Pseudomonas phage vB_PsyM_KIL1
62	44647	45072	426	+	hypothetical protein vB_PsyM_KIL3_0083	1.87e-80	82.27	Pseudomonas phage vB_PsyM_KIL3
63	45038	45241	204	+	hypothetical protein phiPsa315_091	1.31e-32	81.25	Pseudomonas phage phiPsa315
64	45222	45653	432	+	hypothetical protein BH774_gp119	1.15e-79	77.931	Pseudomonas phage vB_PsyM_KIL1

65	45797	46099	303	+	hypothetical protein BH774_gp117	3.16e-60	98.925	Pseudomonas phage vB_PsyM_KIL1
66	46112	46432	321	+	hypothetical protein BH774_gp116	5.37e-66	93.333	Pseudomonas phage vB_PsyM_KIL1
67	47591	46449	1143	-	putative RNA ligase	0	85.263	Pseudomonas phage vB_PsyM_KIL2
68	47901	47608	294	-	hypothetical protein BH774_gp114	1.94e-33	61.386	Pseudomonas phage vB_PsyM_KIL1
69	48015	48143	129	+	hypothetical protein			
70	48291	48605	315	+	hypothetical protein PPSC2_34	4.38e-67	92.308	Pseudomonas phage PPSC2
71	48605	48799	195	+	hypothetical protein			
72	48804	49235	432	+	hypothetical protein VCM_00078	1.98e-43	55.385	Pseudomonas phage VCM
73	49232	49444	213	+	hypothetical protein BH774_gp111	7.09e-34	81.429	Pseudomonas phage vB_PsyM_KIL1
74	49441	49674	234	+	hypothetical protein			
75	49677	49871	195	+	hypothetical protein FDI83_gp117	1.78e-26	74.603	Pseudomonas phage vB_PsyM_KIL4
76	49966	50583	618	+	hypothetical protein M51_121	8.7e-114	80.392	Pseudomonas phage M5.1
77	51015	51356	342	+	hypothetical protein PPSC2_42	8.83e-32	50.45	Pseudomonas phage PPSC2
78	51356	51556	201	+	hypothetical protein vB_PsyM_KIL2_0103	1.63e-32	80.303	Pseudomonas phage vB_PsyM_KIL2
79	51534	51686	153	+	hypothetical protein BH774_gp104	1.64e-08	87.805	Pseudomonas phage vB_PsyM_KIL1
80	51679	52026	348	+	hypothetical protein REC_124	7.7e-50	67.241	Pseudomonas phage REC
81	52023	52190	168	+	hypothetical protein FDI83_gp109	2.96e-26	87.273	Pseudomonas phage vB_PsyM_KIL4

82	52187	54064	1878	+	putative DNA primase/helicase	0	93.821	Pseudomonas phage vB_PsyM_KIL1
83	54122	56416	2295	+	DNA polymerase	0	80.313	Pseudomonas phage REC
84	56611	56874	264	+	hypothetical protein M51_127	1.23e-21	59.77	Pseudomonas phage M5.1
85	56871	57062	192	+	hypothetical protein			
86	57049	57234	186	+	hypothetical protein BH774_gp097	3.09e-23	68.333	Pseudomonas phage vB_PsyM_KIL1
87	57267	57689	423	+	hypothetical protein VCM_00065	4.05e-40	49.359	Pseudomonas phage VCM
88	57797	58804	1008	+	hypothetical protein REC_132	0	90.093	Pseudomonas phage REC
89	58858	58977	120	+	hypothetical protein BH774_gp093	2.15e-07	87.179	Pseudomonas phage vB_PsyM_KIL1
90	58989	59240	252	+	hypothetical protein BH774_gp091	1.29e-49	98.795	Pseudomonas phage vB_PsyM_KIL1
91	59294	60331	1038	+	hypothetical protein VCM_00060	0	90.725	Pseudomonas phage VCM
92	60328	60543	216	+	hypothetical protein CF96_gp104	1.1e-06	38.776	Pseudomonas phage phiPsa374
93	60536	61090	555	+	hypothetical protein VCM_00058	4.2e-111	84.239	Pseudomonas phage VCM
94	61090	61230	141	+	hypothetical protein			
95	61235	61591	357	+	hypothetical protein VCM_00057	5.05e-58	76.271	Pseudomonas phage VCM
96	61588	61803	216	+	hypothetical protein			
97	61800	61970	171	+	hypothetical protein			
98	61967	62740	774	+	hypothetical protein vB_PsyM_KIL5_0122	4.7e-178	92.969	Pseudomonas phage vB_PsyM_KIL5
99	62753	63001	249	+	hypothetical protein phiK7A1_123	6.83e-14	72.093	Pseudomonas phage phiK7A1
100	63053	63265	213	+	putative baseplate protein	9.3e-169	88.538	Pseudomonas phage vB_PsyM_KIL1

101	63262	63414	153	+	hypothetical protein BH774_gp084	4.22e-22	84	Pseudomonas phage vB_PsyM_KIL1
102	63411	63623	213	+	hypothetical protein BH774_gp083	1.64e-30	85.075	Pseudomonas phage vB_PsyM_KIL1
103	63616	64167	552	+	hypothetical protein phiPsa315_124	7.68e-62	75.385	Pseudomonas phage phiPsa315
104	64261	65181	921	+	flavin-dependent thymidylate synthase	1.3e-173	76.038	Pseudomonas phage REC
105	65162	65602	441	+	HNH homing endonuclease	4.3e-105	100	Pseudomonas phage PHB09
106	65589	65801	213	+	hypothetical protein			
107	65794	66129	336	+	hypothetical protein CF96_gp095	6.95e-70	89.189	Pseudomonas phage phiPsa374
108	66139	67194	1056	+	ribonucleotide-diphosphate reductase subunit beta	0	90.462	Pseudomonas phage VCM
109	67191	68921	1731	+	ribonucleoside-diphosphate reductase NrdZ	0	95.486	Pseudomonas phage VCM
110	68989	69180	192	+	hypothetical protein PPSC2_68	3.8e-12	53.226	Pseudomonas phage PPSC2
111	69180	69353	174	+	hypothetical protein vB_PsyM_KIL2_0136	4.98e-28	85.965	Pseudomonas phage vB_PsyM_KIL2
112	69350	69544	195	+	hypothetical protein REC_150	4.73e-31	82.258	Pseudomonas phage REC
113	69544	69876	333	+	hypothetical protein BH774_gp072	2.19e-65	84.404	Pseudomonas phage vB_PsyM_KIL1
114	69879	70049	171	+	hypothetical protein vB_PsyM_KIL2_0139	1.87e-28	87.5	Pseudomonas phage vB_PsyM_KIL2
115	70042	70179	138	+	hypothetical protein FDI83_gp076	4.72e-20	91.111	Pseudomonas phage vB_PsyM_KIL4
116	70189	70413	225	+	putative glutaredoxin	2.22e-40	87.838	Pseudomonas phage vB_PsyM_KIL1

117	70425	70838	414	+	hypothetical protein FDI83_gp074	6.91e-79	84.672	Pseudomonas phage vB_PsyM_KIL4
118	70835	71275	441	+	hypothetical protein PN09_139	4.69e-71	71.918	Pseudomonas phage PN09
119	71290	71727	438	+	hypothetical protein phiK7A1_138	5.8e-51	56.863	Pseudomonas phage phiK7A1
120	71741	72154	414	+	hypothetical protein VCM_00037	1.19e-41	56.204	Pseudomonas phage VCM
121	72154	72420	267	+	hypothetical protein psageK4_148	1.75e-17	51.111	Pseudomonas phage psageK4
122	72496	72825	330	+	hypothetical protein PPSC2_84	3.88e-25	41.085	Pseudomonas phage PPSC2
123	72843	73007	165	+	hypothetical protein PPSC2_83	2.0e-10	48.837	Pseudomonas phage PPSC2
124	73009	74040	1032	+	hypothetical protein phiPsa381_146	0	78.761	Pseudomonas phage phiPsa381
125	74024	74230	207	+	hypothetical protein			
126	74247	74711	465	+	hypothetical protein BH774_gp067	7.27e-79	75.51	Pseudomonas phage vB_PsyM_KIL1
127	74789	74929	141	+	hypothetical protein VCM_00030	3.86e-14	71.739	Pseudomonas phage VCM
128	74931	75326	396	+	hypothetical protein PPSC2_89	3.25e-80	86.154	Pseudomonas phage PPSC2
129	75323	75565	243	+	hypothetical protein phiPsa315_146	2.78e-18	51.724	Pseudomonas phage phiPsa315
130	75562	75891	330	+	hypothetical protein M51_165	7.04e-50	78.182	Pseudomonas phage M5.1
131	75893	76108	216	+	hypothetical protein M51_166	9.26e-34	76.056	Pseudomonas phage M5.1
132	76105	76338	234	+	hypothetical protein BH774_gp060	2.32e-38	76.623	Pseudomonas phage vB_PsyM_KIL1
133	76338	76484	147	+	hypothetical protein BH774_gp059	2.73e-21	91.667	Pseudomonas phage vB_PsyM_KIL1
134	76567	77523	957	+	hypothetical protein CF96_gp068	2.5e-108	76.168	Pseudomonas phage phiPsa374
135	77520	77714	195	+	hypothetical protein BH774_gp058	6.42e-37	92.188	Pseudomonas phage vB_PsyM_KIL1
136	77931	78155	225	+	hypothetical protein			
137	78468	78274	195	-	hypothetical protein			

138	78726	79751	1026	+	hypothetical protein vB_PsyM_KIL2_0154	0	72.365	Pseudomonas phage vB_PsyM_KIL2
139	79820	80197	378	+	hypothetical protein FDG98_gp072	1.06e-19	44.531	Pseudomonas phage pf16
140	80200	80607	408	+	hypothetical protein CNR37_00109	8.15e-21	39.85	Pseudomonas phage ventosus
141	80638	80979	342	+	hypothetical protein BH774_gp053	8.56e-57	87.379	Pseudomonas phage vB_PsyM_KIL1
142	81026	81274	249	+	hypothetical protein BH774_gp050	1.18e-19	86.957	Pseudomonas phage vB_PsyM_KIL1
143	81276	81461	186	+	hypothetical protein	4.7e-15	58.182	Myoviridae sp.
144	81461	81586	126	+	hypothetical protein			
145	81606	81881	276	+	hypothetical protein			
146	82047	82163	117	+	hypothetical protein	1.41e-18	94.737	Pseudomonas phage vB_PsyM_KIL1
147	82166	82546	381	+	hypothetical protein BH774_gp048	4.81e-77	91.2	Pseudomonas phage vB_PsyM_KIL1
148	82619	82912	294	+	hypothetical protein FDG97_gp030	1.61e-41	69.697	Pseudomonas phage phiPMW
149	83011	83337	327	+	hypothetical protein	8.76e-31	59.574	Siphoviridae sp.
150	83381	83731	351	+	hypothetical protein CF96_gp058	4.16e-62	78.378	Pseudomonas phage phiPsa374
151	83719	83850	132	+	hypothetical protein BH774_gp045	3.54e-21	95.349	Pseudomonas phage vB_PsyM_KIL1
152	83921	84142	222	+	hypothetical protein			
153	84224	84616	393	+	hypothetical protein PPSC2_105	3.4e-57	62.963	Pseudomonas phage PPSC2
154	84647	85072	426	+	hypothetical protein FDI83_gp045	1.18e-47	57.931	Pseudomonas phage vB_PsyM_KIL4
155	85069	85206	138	+	hypothetical protein			

156	85203	85379	177	+	hypothetical protein BH774_gp043	2.72e-27	87.037	Pseudomonas phage vB_PsyM_KIL1
157	85373	85711	339	+	NADH oxidase H2O-forming	2.94e-15	43.373	Siphoviridae sp.
158	85715	85924	210	+	hypothetical protein			
159	86029	86163	135	+	hypothetical protein			
160	86326	86571	246	+	hypothetical protein psageK4_179	2.38e-08	38.554	Pseudomonas phage psageK4
161	86643	86798	156	+	hypothetical protein			
162	86860	87054	195	+	hypothetical protein			
163	87075	87359	285	+	hypothetical protein			
164	87431	87589	159	+	hypothetical protein			
165	89204	88977	228	-	hypothetical protein			
166	89406	89206	201	-	hypothetical protein FDI83_gp003	2.79e-33	81.818	Pseudomonas phage vB_PsyM_KIL4
167	89653	89483	171	-	hypothetical protein VCM_00184	7.10e-18	67.308	Pseudomonas phage VCM
168	89853	89653	201	-	hypothetical protein			
169	90038	89850	189	-	hypothetical protein vB_PsyM_KIL2_0004	7.55e-26	70.492	Pseudomonas phage vB_PsyM_KIL2
170	90316	90107	210	-	hypothetical protein BH774_gp005	2.14e-17	68.657	Pseudomonas phage vB_PsyM_KIL1
171	90336	90458	123	+	hypothetical protein			
172	90682	90500	183	-	hypothetical protein			
173	90869	90651	219	-	hypothetical protein			
174	91044	90862	183	-	hypothetical protein			
175	91487	91041	447	-	hypothetical protein MT_57028	1.05e-56	61.745	Pseudomonas phage phiPto-bp6g

176	91609	91484	126	-	hypothetical protein vB_PsyM_KIL5_0008	4.75e-19	90.244	Pseudomonas phage vB_PsyM_KIL5
177	91887	91609	279	-	hypothetical protein BH774_gp008	6.67e-57	90.217	Pseudomonas phage vB_PsyM_KIL1
178	92351	91875	477	-	peptide chain release factor	4.59e-74	74.214	Pseudomonas phage M5.1
179	92638	92435	204	-	hypothetical protein BH774_gp009	2.23e-35	86.567	Pseudomonas phage vB_PsyM_KIL1
180	92823	92635	189	-	hypothetical protein BH774_gp010	8.32e-34	88.71	Pseudomonas phage vB_PsyM_KIL1
181	93010	92816	195	-	hypothetical protein VCM_00176	5.75e-28	77.049	Pseudomonas phage VCM
182	93183	93007	177	-	hypothetical protein BH774_gp011	1.50e-21	81.132	Pseudomonas phage vB_PsyM_KIL1
183	93518	93180	339	-	hypothetical protein REC_12	1.44e-53	74.038	Pseudomonas phage REC
184	93967	93632	336	-	hypothetical protein BH774_gp013	3.80e-60	80.734	Pseudomonas phage vB_PsyM_KIL1
185	94214	93969	246	-	hypothetical protein REC_19	3.10e-36	71.25	Pseudomonas phage REC
186	94795	94214	582	-	DNA recombination-mediator protein A	3.41e-114	82.199	Pseudomonas phage VCM
